# Targeting the mTOR Pathway with Natural Products: A Potential Therapeutic Approach for Autism Spectrum Disorders

**DOI:** 10.2174/011570159X388575251126041202

**Published:** 2026-03-10

**Authors:** Tayebeh Noori, Seyede Zahra Hosseini, Seyede Darya Alavi, Antoni Sureda, Samira Shirooie

**Affiliations:** 1 Pharmaceutical Sciences Research Center, Health Institute, Kermanshah University of Medical Sciences, Kermanshah, Iran;; 2 Student Research Committee, Faculty of Pharmacy, Kermanshah University of Medical Sciences, Kermanshah, Iran;; 3 Research Group on Community Nutrition and Oxidative Stress (NUCOX) and Health Research Institute of Balearic Islands (IdISBa), University of Balearic Islands-IUNICS, Palma de MallorcaE-07122, Balearic Islands, Palma, Spain;; 4 CIBER Fisiopatología de la Obesidad y Nutrición (CIBEROBN), Instituto de Salud Carlos III (ISCIII), Madrid, 28029, Spain

**Keywords:** Autism spectrum disorders, natural products, mTOR, neurodevelopmental disorders, ASD, natural products

## Abstract

Autism spectrum disorders (ASD) are complex neurodevelopmental conditions characterized by a diverse range of challenges and strengths in social communication, behavior, and sensory processing, with wide variation in presentation and severity among individuals. Treatment for ASD symptoms typically includes stimulants, modafinil, atomoxetine, antidepressants, antipsychotics, and natural products. The mechanistic target of rapamycin (mTOR), a serine/threonine kinase, is dysregulated in many human diseases, leading to abnormal synapse formation, neuroinflammation, and mitochondrial dysfunction. Natural products, due to their wide range of bioactive compounds and relatively low toxicity, have emerged as promising modulators of this pathway. Several phytochemicals-including curcumin, resveratrol, and epigallocatechin gallate-have demonstrated the ability to modulate mTOR signaling and improve neurobehavioral outcomes in preclinical models of ASD. mTORC1 and mTORC2 are key regulators of cellular processes, with mTORC1 being largely sensitive to nutrient availability and mTORC2 playing a crucial role in cell growth, proliferation, motility, and autophagy, which is modulated by the PI3K/Akt/mTOR pathway. Future research should examine the relationship between autism and the mTOR signaling pathway, as well as the phytochemicals that may contribute to improving the condition through this pathway.

## 1 INTRODUCTION

Autism spectrum disorders (ASD) are a heterogeneous group of neurodevelopmental syndromes characterized clinically by deficits in social interaction, communication, and repetitive or stereotyped behaviors, often accompanied by varying degrees of sensory impairments and other comorbidities [1]. The global prevalence of ASD is approximately 1%, with one in every 160 children affected worldwide. Autism is more common in males than females, and comorbidities are highly prevalent, with over 70% of individuals experiencing concomitant illnesses [2]. Individuals with ASD exhibit distinct cognitive profiles, including impaired social cognition and perception, executive dysfunction, and atypical perceptual and information-processing patterns. At the system level, these traits are supported by abnormal brain development [2]. The inability to interpret social signals and guide appropriate behaviors may explain the social impairments observed in ASD. The importance of social signal interpretation in social interactions is demonstrated by physiological reactions triggered by potentially threatening situations, such as proximity to others, which help maintain personal boundaries during social encounters [3, 4].

The pathophysiology of ASD is complex and not fully understood. Research indicates that a combination of genetic and environmental factors, particularly those influencing prenatal and early-life development, contributes to the condition [1]. mTOR, a serine/threonine kinase, serves as a master regulator of cellular metabolism. It regulates cell growth and proliferation in response to diverse signals, and its signaling pathway is deregulated in numerous human diseases [5]. Preclinical research on ASD has identified behavioral abnormalities and elevated mTORC1 activity in the brain. However, these effects were reversed with rapamycin, an mTORC1 inhibitor [6]. Disruption of the mTOR signaling cascade leads to downregulation of protein synthesis at dendritic spines, resulting in reduced translation in neuronal cells [6]. Aberrant signaling in regions such as the cerebellum, cerebral cortex, hippocampus, and amygdala contributes to improper synapse formation, glial cell overexpression, neuroinflammation, oxidative stress, and mitochondrial dysfunction [7, 8]. In individuals with ASD, brain studies have revealed altered dendritic spine architecture, abnormal synaptic plasticity, and impaired synaptic homeostasis [9].

Although medications such as antidepressants, antipsychotics, anticonvulsants, and stimulants have shown some efficacy in managing symptoms of ASD, they are unable to fully address its core features. Consequently, the search for safe and effective treatments has increased interest in natural plant-based products with therapeutic potential. Herbal remedies, which tend to have fewer side effects, may offer a safer alternative to mitigate clinical symptoms of ASD [10].

Natural products have long served as a source of disease-modifying compounds. Numerous phytoconstituents exert therapeutic effects on neurodegenerative conditions by targeting distinct cellular and molecular mechanisms, including oxidative stress, inflammatory pathways, and apoptotic processes. Plant-derived bioactive molecules have shown promising preclinical evidence in modulating the PI3K/Akt/ mTOR pathway, suggesting therapeutic potential in the treatment of ASD. However, clinical studies confirming their efficacy and clarifying their pharmacological mechanisms in humans remain limited [4].

This literature review aims to examine key plant-derived natural substances that have shown promise as safe and effective treatments for autism.

## 2 SEARCH STRATEGY AND METHODOLOGY

This study applied a systematic approach to identify relevant literature on the effects of natural compounds on the mTOR pathway in Autism Spectrum Disorders (ASD). Searches were conducted in PubMed, Scopus, Web of Science, ScienceDirect, and Google Scholar for published articles. Keywords included “mTOR pathway,” “autism spectrum disorder,” “natural compounds,” and related terms. Both original research and review articles in English were included, while editorials, case reports, and abstracts were excluded. After removing duplicates, titles and abstracts were screened for relevance, followed by full-text review to assess quality and findings. Studies focusing on the modulation of mTOR by natural products in ASD were selected. The results were synthesized narratively to highlight key findings and research gaps.

## 3 MTOR: TYPES AND THEIR UPSTREAM AND DOWNSTREAM PATHWAYS

The yeast *Saccharomyces cerevisiae*, which contains the genes TOR1 and TOR2 that produce the Tor1 and Tor2 isoforms, was the first organism found to contain the kinase TOR [11]. The kinase domain, located in the C-terminal half of mTOR, shares sequence similarities with the catalytic domain of phosphatidylinositol 3-kinase (PI3K) [12]. mTOR exists as two protein complexes: mTOR Complex 1 (mTORC1) and mTOR Complex 2 (mTORC2) [13, 14]. Each of these multi-component complexes performs a variety of physiological and pathological functions, including macromolecule synthesis, maintenance of homeostasis, cytoskeletal remodeling, angiogenesis, stress responses, and autophagy. Because mTOR plays a crucial role in cell proliferation and differentiation, its dysfunction contributes to the development and progression of cancer [15, 16].

The components of mTORC1 include Raptor (Regulatory-associated protein of mTOR, essential for complex activation), Deptor (DEP domain-containing mTOR-interacting protein), mLST8 (mammalian lethal with Sec13 protein, also known as GβL), PRAS40 (Proline-Rich Akt Substrate of 40 kDa, a negative regulator of mTORC1), and Tti1 (Tel2-interacting protein 1) [5, 17]. In contrast, mTORC2 consists of mLST8, mSIN1 (mammalian stress-activated protein kinase-interacting protein), Rictor (Rapamycin-insensitive companion of mTOR), Deptor, and Protor (Protein observed with Rictor 1 and 2) [18]. Deptor inhibits substrate binding and negatively regulates both mTORC1 and mTORC2. These two complexes have distinct substrate preferences and downstream signaling pathways, allowing them to regulate different cellular functions [19].

When activated, mTORC1 promotes processes such as ribosome biogenesis, suppression of autophagy, and mRNA translation through the phosphorylation of 4E-BP1 and p70S6 kinase. mTORC2, on the other hand, modulates cytoskeletal dynamics and activates SGK1 phosphorylation to regulate cell growth, motility, and protein kinase C activity [20]. mTORC1 also regulates lysosome biogenesis, protein synthesis, autophagy, and energy metabolism in response to cues such as glucose, amino acids, growth factors, and metabolic signals [21]. AMP-activated protein kinase (AMPK) directly phosphorylates Raptor to inhibit mTORC1 under conditions of low cellular energy, thereby inactivating the complex [22]. mTORC2, unlike mTORC1, is nutrient-insensitive and is primarily involved in cell growth, proliferation, and motility [23, 24].

Autophagy is modulated by the PI3K/Akt/mTOR pathway, where PI3K and Akt act as upstream signaling molecules of mTOR [25, 26]. TSC, triggered by PI3K/Akt signaling, promotes mTORC1 activity, while phosphatase and tensin homolog (PTEN), a PI3K antagonist, serves as a key regulator of input signals [27-31]. Akt positively regulates mTORC1, and mTORC2-mediated phosphorylation of Akt enhances mTORC1 activity, ultimately suppressing autophagy [25].

## 4 AUTISM AND ITS RELATED RISK FACTORS

The pathophysiology of ASD remains poorly understood. Multiple factors appear to contribute to the development of this condition, which is considered a complex disorder arising from a combination of genetic, environmental, and immunological influences. Among the potential mechanisms, excitotoxicity and oxidative stress are thought to represent key points of convergence for these risk factors [32].

### 4.1 Genetic Factors

ASD tends to run in families and can manifest through various inheritance patterns and causative genetic variants [32]. The asymmetrical sex distribution, higher incidence in siblings, greater concordance in monozygotic twins, and increasing risk of ASD with greater familial relatedness all suggest a strong genetic influence in its development. The estimated risk of ASD is approximately 59% in monozygotic twins, 13% in full siblings and dizygotic twins, 9% in maternal half-siblings, 7% in paternal half-siblings, and 3% in cousins [32].

Syndromic and non-syndromic autism are two possible manifestations of monogenic autism, which is caused by a single gene mutation. This disorder may result from single nucleotide polymorphisms (SNPs), copy number variations (CNVs), or mutations in genomic DNA. These mutations can interfere with synaptic transmission, plasticity, and signaling pathways. They can also occur in the context of genetic disorders that frequently cause severe intellectual disabilities, such as Angelman syndrome, tuberous sclerosis, and fragile X syndrome [33, 34].

It has been reported that the pathogenetic mechanisms of ASD involve mutations in genes that regulate components of signaling cascades, including the mTOR pathway and the RAS system [35].

### 4.2 Epigenetic Factors

The environment in which a gene is expressed plays a significant role in epigenetic changes. Epigenetics, therefore, refers to the interplay between susceptible genes and environmental stimuli [36]. Autism development is linked to prenatal and postnatal exposure to various pollutants, heavy metals, radiation, and tobacco smoke. These environmental factors induce epigenetic modifications in genes, contributing to ASD development. Prenatal exposure to anti-epileptic medications and bisphenol A has been shown to affect receptor genes, promoting the emergence of ASD phenotypes. Similarly, post-natal exposure to VPA, estradiol, and citalopram has been implicated in ASD pathogenesis. Furthermore, vitamin D deficiency during gestation increases the risk of ASD in children. Epigenetic mechanisms, such as DNA methylation and histone modifications, can also contribute to ASD pathogenesis (Table **1**).

## 5 MTOR AND AUTISM

Megalocephaly, axonal dysregulation, altered neuron size, disrupted connectivity of neuronal circuits, abnormal proliferation of cerebral cells, enhanced protein synthesis, and variations in dendritic spine density across different brain regions are among the characteristics observed in the human brain due to altered signaling pathways [37]. mTOR plays a significant role in several signaling pathways, acting as a central hub that regulates key physiological processes associated with autism [38]. The mTOR pathway regulates synaptogenesis, corticogenesis, and other neuronal processes in the brain [38]. Evidence suggests that autism may have a biological basis in the Akt/mTOR pathway, which governs protein translation at dendritic spines [39, 40]. Mutations in genes encoding components of the Akt/mTOR cascade are linked to conditions with higher rates of autistic traits. Reduced mTOR activity can adversely affect dendritic spines, contributing to autistic phenotypes [39]. Abnormalities in the mTOR pathway may disrupt spine formation and function, potentially leading to autism in affected individuals [4].

Neurogenesis, corticogenesis, and synaptogenesis represent interconnected molecular pathways critical to neurodevelopment. Adult neurogenesis, the generation of new neurons in brain regions such as the hippocampus and olfactory bulb, plays an essential role in brain function and is crucial for the differentiation of functional neurons from neural stem cells (NSCs) and neural precursors [41]. Embryonic neurogenesis includes corticogenesis, the process that gives rise to the mammalian neocortex. This process is necessary for the development of the six cortical layers, during which neuronal migration begins in the ventricular zone and proceeds to its final destination in the brain [42]. The final stage of neural development, known as synaptogenesis, involves the establishment of connections between pre- and postsynaptic domains in target neurons. This stage also includes the initiation and regulation of synaptic growth through the mTOR and Wnt signaling pathways. Following synaptogenesis, the balance between excitatory and inhibitory synapses (E/I ratio) is crucial for the formation of functional neuronal circuits and for the regulation of synaptic output [43]. Disruptions in these essential regulatory mechanisms have been associated with various neurodegenerative and cognitive disorders, including ASD, Parkinson's disease, Alzheimer's disease, Huntington's disease, amyotrophic lateral sclerosis, and schizophrenia [41].

In ASD, functional hyperconnectivity has been linked to mTOR-related synaptic damage. Evidence suggests that behavioral disturbances and ASD may arise from abnormalities in synaptic pruning, leading to altered brain connectivity. During normal brain development, synaptic pruning refines neural networks by removing unnecessary connections between neurons. Overactivity of the mTOR pathway can impair this mechanism and result in an excessive number of synapses in the brain, which is a common characteristic in individuals with ASD [44]. Additionally, mTOR regulates immune cells that contribute to brain inflammation, including microglia. Aberrant mTOR signaling may exacerbate ASD symptoms by increasing the release of inflammatory molecules and promoting persistent neuroinflammation [45]. Furthermore, this pathway establishes a comprehensive, multiscale translational framework that links developmental synaptopathy with functional pathway disruptions in ASD [46].

Various studies have established a correlation between the absence of FMRP and the disruption of mTORC1 in fragile X syndrome (FXS) [47]. Individuals with FXS exhibit dysregulation characterized by elevated levels of phosphorylated mTOR, S6K1, Akt, and eIF4E. The absence of FMRP also leads to excessive activation of the PI3K/Akt signaling pathway. This dysregulation results in spine abnormalities, reduced synaptic plasticity, and cognitive deficits, primarily due to diminished autophagy and overactivation of mTOR [48]. Genetic abnormalities in the PTEN gene have been identified in some individuals with ASD who present with macrocephaly. PTEN acts as a key suppressor of the PI3K/Akt pathway, exerting regulatory control over downstream mTOR signaling. In animal models, the absence of PTEN causes excessive activation of the PI3K/Akt/mTOR signaling pathway in the hippocampus, resulting in chronic alterations in social behaviors that resemble those observed in individuals with ASD (Fig. **1**) [49-55]. Neuroinflammation also appears to play a significant role in the development of ASD. Multiple studies have reported elevated levels of various cytokines and chemokines in individuals with autism [56]. Cytokines activate signal transduction pathways, including JAK-STAT and PI3K/Akt/mTOR, which regulate a wide range of cellular responses. Recent evidence suggests that microglial activation occurs in the brains of individuals with ASD. Elevated concentrations of the pro-inflammatory chemokine (C-C motif) ligand 5 (CCL5) have been consistently observed in the blood plasma of children diagnosed with ASD [57, 58]. Inhibiting mTOR activity has been shown to reduce CCL5 secretion by human microglia, suggesting that mTOR activation plays a role in regulating CCL5 expression and release. Dysregulation of the Akt/ mTOR signaling pathway can also affect cellular proliferation and cytokine production in the immune system, which may, in turn, negatively influence behavior [58].

## 6 NATURAL MTOR MODULATORS

Many variables or targets must be addressed for the effective treatment of neurological disorders. Although autism is a complex neurodevelopmental condition, growing evidence suggests that inflammation and oxidative stress are common features of ASD. Recent research has highlighted the role of the PI3K/Akt/mTOR pathway in the regulation and potential management of autism. By targeting this signaling pathway, naturally occurring compounds in food, known as phytochemicals, may play a significant role in managing neurodegenerative disorders. These compounds may also serve as therapeutic adjuncts within an individual’s nutritional and therapeutic regimen [59]. This diverse group of substances includes dietary polyphenols such as flavonoids, phytoalexins, phenolic acids, indoles, sulfur-rich compounds, and vitamins (carotenoids) [60]. Their ability to modulate key molecular pathways positions them as promising candidates for complementary therapies in neurological and neurodevelopmental conditions.

### 6.1 Sulforaphane

Sulforaphane (SFN), an isothiocyanate derived from fruits and vegetables such as Brussels sprouts, broccoli, and cabbage, exhibits antioxidant and anti-inflammatory properties [61]. It targets numerous proteins in mammalian cells and disrupts processes that are critical for maintaining cellular homeostasis. SFN specifically inhibits the activity of cytoplasmic histone deacetylase 6 (HDAC6), which causes Akt, a crucial upstream activator of mTOR, to become more acetylated and less catalytically active. This reduction in Akt activation facilitates the downregulation of mTOR signaling. However, it remains unclear whether SFN interacts directly with mTOR complexes (mTORC1 or mTORC2), suggesting that the inhibition of mTOR primarily occurs through upstream modulators [15, 62].

SFN also inhibits the function of HDAC6 and reduces the enzymatic activity of Akt, contributing further to the suppression of mTOR activity. This effect was observed in a study by Wiczk *et al*., in which 40 μM of SFN was applied to PC-3 and SKBR-3 cells [63, 64]. Several additional experiments have demonstrated SFN's ability to regulate autophagy and mTOR signaling. For example, in a study that used MDA MB 231, MCF-7, MDA MB 468, and SKBR-3 cells, SFN administered at concentrations of 5, 10, 20, 30, or 40 μM significantly reduced Akt and S6K1 kinase phosphorylation, inhibited protein synthesis, and induced autophagy. These findings strongly suggest that SFN, by targeting downstream components of the PI3K-Akt-mTOR-S6K1 pathway, holds potential as a therapeutic agent for the treatment of breast tumors [65-67].

In a clinical trial, Singh *et al*. demonstrated that SFN treatment (50 µmol of SFN for individuals under 100 lb, 100 µmol for 101 to 199 lb, and 150 µmol for over 200 lb) substantially improved social impairments associated with ASD. Due to its ability to address several ASD-related dysfunctions, including oxidative stress, reduced antioxidant capacity, impaired glutathione synthesis, diminished mitochondrial activity, and neuroinflammation, dietary SFN has been investigated as a potential therapeutic agent [67]. Multiple studies also suggest that SFN may regulate autophagy and mTOR signaling, reinforcing its potential role in managing ASD and other related disorders [68-70].

According to a randomized controlled trial (RCT) conducted on young males with moderate to severe ASD, daily intake of SFN can significantly improve verbal communication, disruptive behavior, and social interaction without causing significant toxicity [67]. The Social Responsiveness Scale (SRS), which measures social responsiveness, showed notable improvements in children with ASD treated with sulforaphane in another open-label trial [71]. Additionally, it has been demonstrated that cauliflower extracts enriched with SFN can significantly improve social interactions, aberrant behaviors, and verbal behaviors in young individuals with autism spectrum disorders [72].

### 6.2 Naringenin

Naringenin (NR) is a bioflavonoid known as 4,5,7-trihydroxyflavonone. Its pharmacological properties include hepatoprotective, antidiabetic, anticancer, anticholesterolemic, wound healing, antiatherosclerotic, hypolipidemic, anti-inflammatory, and antioxidant effects [73]. NR activates antioxidant signaling pathways through multiple mechanisms. It mitigates the toxic effects of free radicals by increasing the mRNA and protein levels of nuclear factor erythroid-related factor 2 (Nrf2), a transcription factor associated with antioxidant biosynthesis [74].

NR modulates various antioxidant enzyme parameters, and non-enzymatic components such as glutathione reductase (GSH), glutathione S-transferase (GST), superoxide dismutase (SOD), glutathione peroxidase (GPx), paraoxonase (PON), catalase (CAT), malondialdehyde (MDA), and total antioxidant capacity (TAC) in different brain regions, including the cortex, hippocampus, striatum, and whole brain, thereby exerting potent neuroprotective effects. Naringenin can improve cognitive deficits and neuronal damage by enhancing antioxidative capacity [75].

It has been reported that intraperitoneal injection of naringenin exerts neuroprotective effects through anti-inflammatory activity and induction of glial-derived neurotrophic factor (GDNF), which supports the survival of dopaminergic neurons in animal models of Parkinson's disease [76]. NR has also been shown to play an important role in reducing neuropsychopathology associated with ASD, including repetitive behaviors, hyperlocomotion, anxiety-like behaviors, sensorimotor dysfunction, and impaired social interaction [77]. Additionally, NR demonstrates antiproliferative, antihyperlipidemic, and antidepressant activities, along with beneficial effects on cognitive dysfunction and metabolic disorders, providing a pharmacological basis for its potential use in preventing and treating ASD [78, 79].

In rats with ASD induced by propanoic acid, oral administration of naringenin for 29 days resulted in significant behavioral and biochemical improvements [80]. Moreover, by regulating AKT1 target proteins, NR can maintain cellular activity and modulate apoptosis [81]. To prevent apoptosis in autism and reduce aberrant social behavior in mice, NR can simultaneously inhibit AKT1 phosphorylation, enhance protein synthesis and gene transcription, and stimulate Akt-mTOR signaling [82, 83].

### 6.3 Apigenin

Apigenin is a natural flavonoid predominantly found in citrus fruits in a glycosylated form. It is well-known for its antioxidant, anticancer, and anti-inflammatory effects [84, 85]. Zhang *et al*. demonstrated in an *in vivo* study that apigenin, at a dose of 60 mg/kg, exhibited antidepressant properties [86]. These effects were achieved by inducing autophagy through the AMPK/mTOR pathway in mice subjected to restraint stress. Similarly, Lu *et al*. reported that 40 μM apigenin promotes autophagosome formation and increases LC3-II expression while reducing mTOR protein levels and P62 protein expression. Their study, conducted on HepG2 cells, concluded that the hypolipidemic effects of apigenin are linked to autophagic lipid degradation [87].

In another study, Abd Elazeem *et al*. investigated the effects of apigenin on VPA-induced autism in 70 male Wistar albino rats. Their findings revealed that apigenin (40 mg/kg/day) can alleviate oxidative stress and apoptosis while modulating autophagy and inhibiting mTOR signaling. These mechanisms contributed to a notable improvement in histopathological outcomes and behavioral impairments. Additionally, the combination of apigenin with risperidone yielded superior results compared to either treatment alone, suggesting a synergistic therapeutic benefit [88].

### 6.4 Berberine

Berberine is a herbal supplement beneficial for the treatment of mood disorders [89]. It is an isoquinoline alkaloid found in the roots, rhizomes, stems, and bark of numerous plants, including *Coptis chinensis* and *Berberis vulgaris* [90]. This compound exhibits antimicrobial, anti-protozoan, anti-diarrheal, anticancer, anti-obesity, and anti-inflammatory properties. Moreover, it plays a role in the regulation of neurotransmitters and their receptors in the central nervous system [91, 92]. In animal models, berberine has been shown to exert neuroprotective and cardiovascular protective effects [93]. It may reduce oxidative stress by stimulating the expression of SOD, nicotinamide adenine dinucleotide phosphate (NADPH) oxidase, and uncoupling protein 2 (UCP2), potentially mediated through the SIRT1/FOXO or AMPK pathway [94].

In a study conducted by Wang *et al*. on human glioma cell lines (U251 and U87) and athymic male mice, berberine (50 mg/kg/day) reduced p-mTOR levels while increasing AMPK levels. These findings suggest that berberine promotes mTOR-dependent autophagy in glioblastoma multiforme cells [95]. According to an *in vitro* analysis by Li *et al*. on HCT116, SW480, and LOVO colon cancer cell lines, berberine (15 μmol/L) activated AMPK, a key regulator of metabolic pathways, and inhibited mTOR, a downstream target of AMPK. Additionally, treatment with berberine resulted in the downregulation of 4E-binding protein-1 and p70 ribosomal S6 kinase, which are downstream effectors of mTOR. The inhibition of mTOR activity and p53 phosphorylation by berberine was determined to be AMPK-dependent [96].

Similarly, VPA-induced oxidative stress, behavioral abnormalities, and cerebellar neurodegeneration can be effectively prevented by berberine due to its strong anti-inflammatory and antioxidant properties. It can protect neurons by reducing anxiety-related effects and motor coordination impairments caused by VPA. Berberine therapy was considered beneficial for strengthening Purkinje cells in the cerebellum and enhancing cerebellar-related functions, making it more suitable for treating ASD by restoring oxidative balance [97, 98]. Taken together, berberine has been extensively shown in numerous preclinical studies to exert therapeutic effects on a wide range of central nervous system disorders, including Alzheimer's disease, cerebral ischemia, depression, schizophrenia, epilepsy, anxiety, and autism [91, 99-103].

### 6.5 Curcumin

Curcumin is a natural compound obtained from the rhizome of the turmeric plant. This compound exerts various pharmacological and therapeutic effects through multiple cellular and molecular mechanisms [104, 105]. It offers strong anti-inflammatory and anti-neoplastic properties. It has been discovered that curcumin plays an important role in regulating the mTOR signaling pathway [4]. In male Sprague Dawley rats with autistic characteristics, curcumin (at a maximum dosage of 200 mg/kg) has been demonstrated to decrease matrix metalloproteinases, oxidative stress, mitochondrial defects, and tumor necrosis factor (TNF) release. Consequently, curcumin has been found to function as a neuropsychopharmacotherapeutic agent in the treatment of ASD [106]. Curcumin has demonstrated antioxidant and radical-scavenging activity *in vitro* and *in vivo*, and can therefore reduce several inflammatory markers in a variety of illnesses [106].

The effectiveness of curcumin in treating TSC in Tsc2 knockout mice was examined by Kuo *et al*. [107]. In Tsc2+/- mice, oral treatment with a solid lipid particle formulation of curcumin inhibits mTOR activity while activating AMPK activity. The mice's recognition memory deficit was also restored by curcumin, indicating a significant therapeutic effect on TSC. In another study, rats with propionic acid (PPA)-induced autism were given curcumin at daily doses of 50, 100, and 200 mg/kg for 28 days, which reduced mitochondrial dysfunction, oxidative-nitrosative stress, TNF-α, and MMP-9, and also improved behavioral outcomes [108].

### 6.6 Resveratrol

Resveratrol, a phytoalexin and polyphenol, is found in the skin of red grapes, red wine, Japanese knotweed, and certain nuts [109]. It possesses a range of biological activities, including anti-inflammatory, anti-cancer, anti-apoptotic, and antioxidant properties [110]. By modulating brain activity, resveratrol has been shown to enhance cognitive functions related to learning, anxiety, depression, and memory [111, 112]. Research conducted by Zhou *et al*. demonstrated that resveratrol may suppress the proliferation of hepatocellular carcinoma cells by downregulating the PI3K/AKT/ mTOR signaling pathway [113]. Moreover, evidence from a study in male rats suggests that stimulating the PI3K/AKT signaling pathway with 30 mg/kg of resveratrol may provide neuroprotection by preventing neuronal death following cerebral ischemia [114]. Recent evidence indicates that the suppression of the PI3K/AKT/mTOR signaling pathway could be a key mechanism underlying apoptosis induction and the inhibition of cell proliferation [115-117]. Furthermore, since resveratrol protects synaptic plasticity, it has neuroprotective properties [61]. Resveratrol has been shown to suppress mTOR in several preclinical investigations [118, 119]. Preclinical research using propanoic acid-induced ASD rat models has shown that administering resveratrol (5, 10, and 15 mg/kg) for 28 days can improve behavioral deficits and reduce levels of TNF-α and IL-6 as inflammatory cytokines in the brain [120]. Additionally, resveratrol supplementation during pregnancy prevented social deficits in a mouse model of autism caused by VPA exposure during pregnancy [121].

### 6.7 Omega 3

Omega-3 fatty acids are powerful anti-inflammatory agents that inhibit the synthesis of immunomodulatory molecules such as cytokines. Consequently, increased omega-3 consumption has consistently been shown to reduce inflammation. Additionally, omega-3 fatty acids have been found to alleviate H. pylori-induced gastric irritation. Docosahexaenoic acid (DHA), a key omega-3 fatty acid, is an essential structural component of the brain and retina. Moreover, omega-3 fatty acid intake has been associated with a reduced risk of macular degeneration [32].

Fish and seafood are rich sources of eicosapentaenoic acid (EPA) and DHA, both of which are long-chain omega-3 fatty acids [122]. During a child’s brain development, maternal polyunsaturated fatty acid (PUFA) levels play a pivotal role in regulating microglial activation and neuroinflammatory pathways. It is hypothesized that abnormal or insufficient PUFA metabolism in children with ASD may lead to increased production of proinflammatory cytokines, heightened oxidative stress, and imbalances in neurotransmitter synthesis and function. Furthermore, omega-3 PUFA deficiency may alter gut microbiota composition, potentially contributing to ASD through disruptions in the gut-brain axis [122, 123]. Shirooie and her colleagues have shown that omega-3 fatty acids, by targeting mTOR, have a positive effect on neurodegenerative diseases [124].

### 6.8 Piperine

Piperine, the primary alkaloid found in black pepper, possesses antioxidant and neuroprotective properties [125]. Recent research in cell lines and animal models has shown that piperine exhibits anti-inflammatory, anticarcinogenic, immunomodulatory, and antioxidant effects [126]. Its anti-apoptotic properties have been reported in neurological conditions such as Alzheimer’s disease [127] and Parkinson’s disease [128]. According to these studies, piperine can improve memory in individuals with these conditions due to its anti-inflammatory and antioxidant activities. In addition, pretreatment with piperine in animal models of these disorders has demonstrated a protective effect against memory impairment [129, 130].

In an *in vivo* study, injecting VPA (400 mg/kg) into 14-day-old mice increased oxidative stress and induced behavioral features resembling those of autistic patients. Piperine was administered concurrently to the VPA-treated animals from postnatal days 13 to 40. This treatment improved ASD-like behavioral characteristics, reduced oxidative stress in the brain, and restored cerebellar histoarchitecture. Additionally, motor deficits were ameliorated. Therefore, piperine demonstrated neuroprotective effects, likely due to its antioxidant properties [131].

### 6.9 Baicalin

Another flavonoid that has several pharmacological activities is baicalin (BAI) [132]. In recent years, research has shown that baicalin has several potential health benefits due to its anti-inflammatory, antioxidant, and neuroprotective effects [92]. BAI's ability to heal the central nervous system (CNS) is strengthened by its distribution throughout the brain [133]. Additionally, the neuroprotective effects of BAI and its aglycone BE have also been studied. By inhibiting neuroinflammation, boosting neuronal regeneration, and decreasing neuronal death, they may provide neuroprotection [134, 135]. By preventing NF-κB activation *via* a variety of signal transductions, including nuclear factor erythroid 2-related factor 2 (Nrf2)/heme oxygenase-1 (HO-1), Toll-like receptor 4 (TLR4), mitogen-activated protein kinase (MAPK), and AMP-activated protein kinase (AMPK), BAI or BE mediates anti-inflammatory effects and ultimately has neuroprotective effects in neurodevelopmental disorders (NDDs) [136]. An *in vivo* study has indicated that BAI enhanced motor development, repetitive behaviors, and social impairments in rats prenatally exposed to VPA modeling, which was performed on gestation day 12.5. *Via* downregulating caspase-3 expression and upregulating SIRT1 in SH-SY5Y cells, BAI prevents neuroinflammation and apoptosis [134]. It has been demonstrated that the nuclear factor κ-light chain enhancer (NF-κB) of activated B cells regulates the release of pro-inflammatory cytokines and the body's reaction to stress [137]. The expression of NF-κB and its phosphorylation at the Ser536 site are significantly altered in animal models as well as in the cerebellum and cortex of individuals with ASD [138].

### 6.10 Chrysophanic Acid

Chrysophanic acid, also known as chrysophanol (CPH), is a natural anthraquinone compound with anti-inflammatory, antioxidant, and neuroprotective properties. It is commonly found in various plants, including *Rheum* species (rhubarb), *Cassia* species, and *Aloe vera* [139, 140]. Studies indicate that CPH exhibits a broad range of biological activities and pharmacological effects, including anti-cancer, anti-depressant, and anti-bacterial properties [141]. Additionally, CPH has been used to treat a variety of neurological illnesses [142, 143] and displays antiviral and anti-inflammatory qualities [144]. Furthermore, CPH may offer a potential treatment option for individuals with autism and other neurodevelopmental disorders as a PI3K/AKT/mTOR inhibitor [140].

The pathological, neuroprotective, and neurochemical effects of chrysophanol at doses of 10 mg/kg and 20 mg/kg were examined in rats with an experimental model of autism induced by propionic acid [143]. The study’s findings suggest that CPH restored altered neurochemical levels and prevented demyelination and other severe pathological changes in the brains of autistic rats. CPH also improved memory, learning, and social interaction deficits. Additionally, it effectively inhibited the PI3K/Akt/mTOR pathway. The summarized information related to the discussed natural products is presented in Table **2**.

## CONCLUSION

This review highlighted the potential of phytochemicals to improve the molecular and behavioral aspects of ASD. A detailed analysis of the pathogenic mechanisms of ASD, particularly through the lens of mTOR signaling, emphasizes the relevance of this pathway in autism research. Given the complexity of ASD etiology, targeting shared molecular disruptions such as mTOR may provide a more unified framework for intervention strategies. Several studies have provided promising evidence that compounds such as sulforaphane, resveratrol, and omega-3 fatty acids may exert beneficial effects on ASD. These compounds are believed to modulate neuroinflammation and oxidative stress, both of which are implicated in ASD pathogenesis. However, these findings remain preliminary because of the lack of large-scale, blinded, and randomized clinical trials. To address this gap, future research should focus on well-designed, controlled trials to validate preclinical findings and establish effective therapeutic strategies. Further integration of clinical and molecular data will be essential to move phytochemical interventions closer to clinical application in ASD.

## STUDY LIMITATIONS

This review is subject to several limitations. As a narrative review, it lacks a systematic methodology, which may introduce selection bias. The included studies vary in design, quality, and scope, limiting the strength and consistency of the conclusions. Furthermore, the absence of quantitative synthesis restricts the ability to compare outcomes directly, and the findings remain dependent on the availability and reliability of published data.

## Figures and Tables

**Fig. (1):**
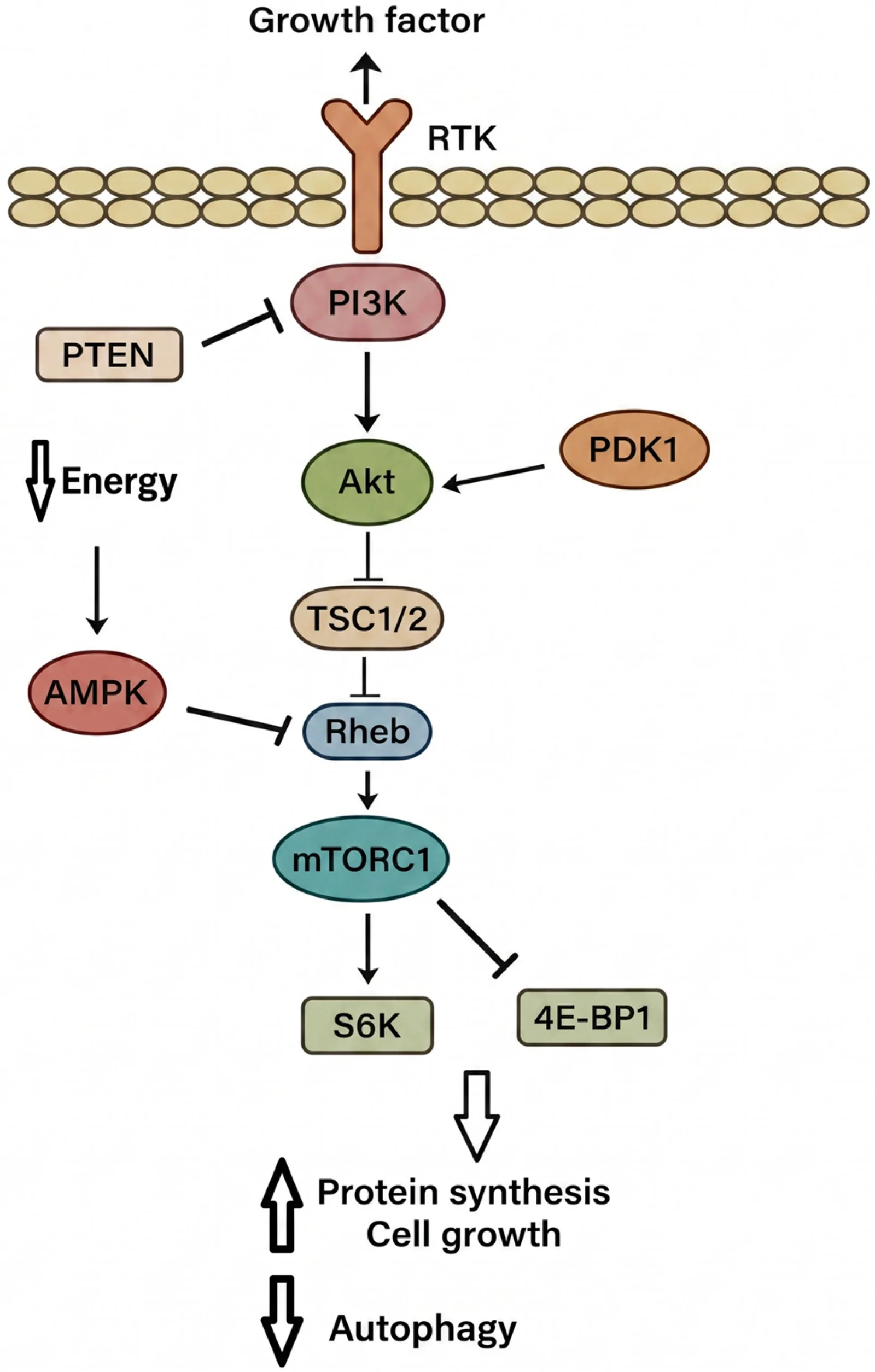
Schematic representation of the PI3K/Akt/mTOR signaling pathway.

**Table 1:** Risk factors in ASD.

**Epigenetic Risk Factors**	
Age	The risk of ASD has been associated with advanced parental age, though the underlying mechanisms remain unclear. The likelihood of developing ASD increases with maternal and paternal age over 40 years, particularly in men, possibly due to enhanced methylation of sperm DNA, which may contribute to a higher risk of autism.
Prenatal Exposure	Prenatal exposure to factors such as changes in sex hormones, maternal overweight, high blood pressure, diabetes, infections (including rubella, cytomegalovirus, and influenza), *in vitro* fertilization (IVF), immune system activity, and ultrasound have all been associated with an increased risk of ASD.
Prenatal risks	Caesarean delivery, low birth weight, poor Apgar score, and hypoxia may all contribute to an increased risk of autism.
Smoking and alcohol	Smoking and alcohol consumption have been associated with neurological, psychiatric, and neurodevelopmental disorders, including ASD comorbidities such as ADHD. Although smoking has been specifically linked to an increased incidence of ASD with intellectual disability (ID), two meta-analyses have found no evidence to support smoking as a general risk factor for ASD.
Vaccine	The link between vaccinations and ASD has been extensively studied. Research indicates that the signs of ASD are often present prior to the administration of the first dose of the MMR vaccine; however, epidemiological evidence does not support a causal relationship between vaccinations and ASD.
Drugs	The potential role of medication use during pregnancy in the development of ASD has been explored evidencing that valproic acid (VPA) is associated with an increased incidence of ASD. Conversely, prenatal exposure to selective serotonin reuptake inhibitors (SSRIs) has not been linked to a heightened risk of ASD.
Nutrition	According to studies, deficiencies in essential nutrients such as vitamin D, L-carnitine, zinc, iron, and copper have been found to significantly impact the likelihood of giving birth to a child with autism.

**Table 2:** Summary of some natural products for the treatment of ASD by targeting the mTOR pathway.

**Natural Product**	**Mechanism**	**Kind of Study**
Sulforaphane	Improves verbal communication, disruptive behavior, and social interaction	Randomized controlled trial
Naringenin	Prevents apoptosis, lessens aberrant social behavior, dramatically restrains AKT1 phosphorylation, enhances protein synthesis and gene transcription, and stimulates signaling of Akt-mTOR	*In vivo*
Apigenin	Alleviates oxidative stress and apoptosis, while also modulating autophagy and inhibiting mTOR signaling	*In vivo*
Berberine	Prevents behavioral abnormalities and cerebellar neurodegeneration. And of course, protects the neurons by lessening the impacts of anxiety and the impairments in motor coordination	*In vivo*
Curcumin	Reduces mitochondrial dysfunction, oxidative-nitrosative stress, TNF-α, and MMP-9, and also improves behavioral results	*In vivo*
Resveratrol	Improves behavioral deficits and reduces the levels of TNF-α and IL-6 as inflammatory cytokines in the brain	*In vivo*
Omega 3	Reduce proinflammatory cytokines, oxidative stress	*In vivo*
Piperine	Improves the behavioral characteristics of the mice with ASD, decreases oxidative stress in the brain, repairs the cerebellar histoarchitecture, and improves their motor deficits	*In vivo*
Baicalin	Enhances motor development, repetitive behaviors, and social impairments	*In vivo*
Chrysophanic acid	enhances memory, learning, and social interaction. And of course, inhibits the PI3K/Akt/mTOR pathway	*In vivo*

## LIST OF ABBREVIATIONS

AMPK: AMP-Activated Protein Kinase
ASD: Autism Spectrum Disorders
BAI: Baicalin
CAT: Catalase
CCL5: (C-C motif) ligand 5
CNS: Central Nervous System
CNVs: Copy Number Variations
CPH: Chrysophanol
Deptor: DEP Domain-containing mTOR-interacting protein
DHA: Docosahexaenoic Acid
EPA: Eicosapentaenoic Acid
FXS: Fragile X Syndrome
GDNF: Glial-Derived Neurotrophic Factor
GPx: Glutathione Peroxidase
GST: Glutathione S-Transferase
HIF: Hypoxia-Inducible Factor
HO-1: Heme Oxygenase-1
MAPK: Mitogen-Activated Protein Kinase
MDA: Malondialdehyde
mLST8: mammalian Lethal with Sec13 Protein, also known as GβL
mTOR: Mechanistic Target of Rapamycin
mTORC1: mTOR Complex 1
mTORC2: mTOR Complex 2
NADPH: Nicotinamide Adenine Dinucleotide Phosphate
NDDs: Neurodevelopmental Disorders
NF-κB: Nuclear Factor κ-Light Chain Enhancer
NR: Naringenin
Nrf2: Nuclear Factor Erythroid-Related Factor 2
NSCs: Neurons From Neural Stem Cells
PI3K: Phosphatidylinositol 3-Kinase
PON: Paraoxonase
PPA: Propionic Acid
PRAS40: Proline-Rich Akt Substrate of 40 kDa, a Negative Regulator of mTORC1
PTEN: Phosphatase and Tensin Homolog
PUFA: Polyunsaturated Fatty Acid
RCT: Randomized Controlled Trial
SFN: Sulforaphane
SNPs: Single Nucleotide Polymorphisms
SOD: Superoxide Dismutase
SRS: Social Responsiveness Scale
TAC: Total Antioxidant Capacity
TLR4: Toll-Like Receptor 4
TNF: Tumor Necrosis Factor
UCP2: Uncoupling Protein 2
